# Access to treatment for Hepatitis C among injection drug users: results from the cross-sectional HOPE IV study

**DOI:** 10.1186/s12939-017-0601-3

**Published:** 2017-06-14

**Authors:** Kyriakos Souliotis, Eirini Agapidaki, Manto Papageorgiou, Niki Voudouri, Xenophon Contiades

**Affiliations:** 1 Department of Social and Education Policy, University of Peloponnese, Faculty of Social and Political Sciences, Damaskinou & Kolokotroni Str, 20100 Corinth, Greece; 2 Health Policy Institute, 36-38, Amaryssias Artemidos Str, 15124 Athens, Greece; 3 Praksis, Independent Non-Governmental Organization, 57 Stournari Str, 10432 Athens, Greece; 4 Centre for European Constitutional Law, Athens, Greece

**Keywords:** Hepatitis C, HCV, Injecting drug users, Greece, Healthcare, Pharmaceuticals, Barriers, Patient access, Recession, Economic crisis

## Abstract

**Background:**

Evidence suggests that Greece is among the European countries with increased trend in HCV prevalence among injecting drug users (IDUs) from 2008 to 2014. Nonetheless, the access of IDUs to treatment for Hepatitis C Virus (HCV) is very limited while the risk of co-infection and transmission remains high. In an effort to better understand the inhibitors to HCV treatment, the present study aimed to investigate the main barriers to access in a sample of IDUs.

**Methods:**

The cross-sectional study was carried out between July and September 2015 using a 23-items questionnaire. Participants were recruited from urban primary services, mobile health vans, community health services, day-care centers as well as during street work, located in Athens, Greece. Inclusion criteria were age above 18 years, understanding and speaking Greek sufficiently, HCV diagnosis, intravenous drug use. Data collection was carried out by health professionals of Praksis, a non-governmental organization. For the comparisons of proportions chi-square and Fisher’s exact tests were used.

**Results:**

The study sample consisted of 101 HCV patients, 68% male. More than 80% of study participants experienced barriers in accessing their doctor and medication during the past 12 months. The most common obstacles in accessing a doctor were “delay in making the appointment and “difficulties in going to the doctor due to health condition or lack of means of transport”. Access to physician or medication was not differed according to gender, but significant differences were found according to economic status and health insurance coverage. 56.1% of participants reported loss or treatment delay due to barriers to treatment. The majority of participants had deteriorated financial status, health status, access to health services and medication, higher financial burden for health services, worse mental health and lower adherence to medical instructions in 2015 compared to 2009.

**Conclusions:**

The findings from the present study revealed that the vast majority of IDUs experience significant barriers in seeking HCV care in Greece, thus highlighting the need for immediate action in this particular area due to the high risk of co-infection and transmission.

## Background

Hepatitis C virus is a liver disease which may cause cirrhosis and cancer if it remains untreated. Injection drug use (IDU) constitutes the leading cause of morbidity for Hepatitis C Virus (HCV) infection in high-income countries [1]. The different surveillance systems adopted in EU-countries do not allow determining the geographical distribution of HCV in European Union although they provide reliable epidemiological data in most cases [2]. It is estimated that the prevalence on HCV is 50 times higher in individuals who inject drugs compared with the general population [3]. In North America, Europe and Australia the prevalence of HCV among injecting drug users (IDUs) ranges from 50 to 90% in all settings [4, 5], while the highest is reported in low and middle income countries like Africa and Asia [6]. The global burden of HCV in injecting drug users is approximately 67% [7].

Recent evidence suggests that IDUs should be included in the treatment of chronic and acute HCV, however the access to treatment for this group is still limited [3, 8]. In previous years, IDUs were excluded from HCV treatment due to concerns of poor adherence, psychiatric co-morbidity and re-infection [9]. Research shows that treatment may effectively address HCV at 95% but the cost of these therapies is substantially high [10]. Limited access to effective HCV treatment contributes to poor health outcomes for IDUs also due to the frequent co-infection of HCV and HIV which in turn increases the mortality risk [11]. Despite the fact that HCV prevalence among IDUs tends to be epidemic especially in younger individuals, their access to treatment is particularly low [3].

According to the international literature, low access to HCV treatment in western countries is attributed to multiple factors emerging at patient’, practitioner’ and healthcare system’ levels. Lack of awareness and low health literacy, hesitation to take medication due to misconceptions about side effects, low rates of compliance, co-morbidity and limited accessibility of treatment locations due to transportation costs (e.g. HIV) are among the key obstacles identified at patient level [12–14]. Furthermore, there are also barriers at the practitioner level which may significantly impede the access to effective treatment for HCV such as the lack of availability and accessibility, limited knowledge and the stigmatization of patients who use drugs [10, 15, 16]. Apart from patient and practitioner’ related factors considered as responsible for the low rates of HCV treatment accessibility, there are also some aspects linked to the healthcare system. For instance, the lack of consensus among countries about the screening and treatment guidelines contributes to the low detection and treatment rates, while in many cases the organizational deficiencies (e.g. limited infrastructure, lack of promotion and funding, long waiting lists), especially in substance use related services and primary care which constitute the main services delivered to IDUs, have resulted in an unfavorable situation towards the access of marginalized groups to HCV treatment [3, 17–20]. Thus, HCV in most western countries often remains under-recognized and untreated, while the injecting drugs users are affected by increased inequalities in access to health and healthcare [19].

Greece is among the European countries with increased trend in HCV prevalence among IDUs from 2008 to 2014 [3, 21]. Apart from the aforementioned barriers which also exist in most European countries, the ongoing economic crisis in Greece makes access to treatment for chronic diseases such as HCV even more challenging [21]. The outbreak of the recession in Greece in 2008 had impacted negatively both the health system and the population’ health outcomes [22, 23]. Before this, many patients sought help to the private health sector. As a result, the public health system was better able to respond to the health needs of the population [24]. One should expect that before the crisis the IDUs should have better access to treatment but misconceptions and stigmatization for this particular group of patients deprived the opportunity to have prompt, appropriate and sufficient access to treatment [25].

However, one year after the onset of the financial crisis, the GDP of the country was substantially decreased while unemployment rates and poverty have been increased [26]. The governments preferred to decrease the public health spending rather to implement the necessary reforms in order to meet the fiscal targets [27]. On the other hand, the income reductions did not allow patients to seek for help at the private sector. Consequently, the national health system had to deal with increased demand and low resources which in turn led to limited availability, accessibility and adequacy of health services [21, 23]. Healthcare access was challenged further for patients due to transportation costs, long waiting lists and financial costs for medication (in case of unemployed patients) [28]. The horizontal budget cuts and the reduced public expenditure on health implemented as a response to the recession have limited the administration of HCV treatment to IDUs in late stages of liver disease [29]. Thus, in 2015 only a low percentage of IDUs have free of charge access to HCV treatment in Greece, while the risk of co-infection and transmission remains high [29].

In this context, the primary objective of the present HOPE IV study was to investigate the main barriers to access to HCV treatment in a sample of injecting drug users, while a secondary aim was to explore if the health outcomes and access to treatment have been altered for this vulnerable patient group since the onset of the economic crisis (for the years 2009 and 2015).

In our previous work, we have endeavored to assess the impact of the Greek economic crisis on access to health and pharmaceutical care of three distinct patient groups, i.e. patients with rheumatoid arthritis, patients with multiple sclerosis and patients with cancer. This work is illustrated in the periodical ‘Health Outcomes of Patient Environment’ (HOPE I, II, III) studies [30–32].

## Methods

### Participants and procedures

The HOPE IV study was carried out between July and September 2015 in order to examine the barriers to access of injecting drug users to HCV treatment. Participants were recruited from urban primary services, mobile health vans (providing rapid tests for HCV, Hep-B, HIV and HCV treatment), community health services for vulnerable groups, day-care centers for homeless people as well as during street work. Inclusion criteria for participation in the study were: age above 18 years, understanding and speaking Greek sufficiently, HCV diagnosis, and intravenous drug use. The study was approved by the Institutional Review Board of the University of Peloponnese and written informed consent was given by all respondents. A convenient sample of 136 potential participants was recruited to participate in the study. Finally, 101 IDUs suffering from HCV met the inclusion criteria and agreed to take part (response rate 74%). Injection drug users in Greece are usually homeless and do not stay in a place for a long time. Therefore, it is not feasible to list all IDUs suffering HCV. The only records available are those from several NGOs doing street work and provide health services to this population. As a consequence, a random and representative sample of IDUs could not be used and thus a convenient sample was preferred.

Data were collected by using a 23-items questionnaire. The tool consisted of three sections: a) 11 items about socio-demographics, b) seven items about access to HCV treatment and healthcare services, c) five items about the current socioeconomic status of the participants and its alteration for the years 2009 and 2015. Close-ended questions, open-ended questions and Likert-type scale items were used. All items were drafted after an exhaustive literature review about the barriers to access to HCV treatment for IDUs. The first draft of the tool consisted of 38 items. A panel of experts (two health researchers, one IDU, three health professionals, two members of HIV/HCV patient associations) was invited to review the first version of the questionnaire. After review the tool was reduced to 23 items. The experts suggested three areas of investigation: a) socio-demographics, b) access to HCV treatment and healthcare services c) socioeconomic status. The final version of the questionnaire was pilot- tested to a small sample of IDUs prior to the final administration.

Data collection was carried out by Praksis health professionals. Praksis is a non-governmental organization based in Athens, which provides social and medical support to vulnerable groups across the country on a voluntary basis. The health professionals of Praksis attended a brief training about the questionnaire administration in order to ensure that the process will be conducted in an appropriate, effective and of high-quality manner. Each health professional approached IDUs during their visits to Praksis street work, in mobile health vans, urban primary and community services, as well as the day-care centers for homeless people and carried out a short-interview with each participant to complete the study questionnaire. At the beginning of each interview, individuals were informed about the voluntarily participation and the anonymity and confidentiality of data.

### Statistical analysis

Quantitative variables are expressed as mean values (SD). Qualitative variables are expressed as absolute and relative frequencies. For the comparisons of proportions chi-square and Fisher’s exact tests were used. All p-values reported are two-tailed. Statistical significance was set at 0.05 and analyses were conducted using SPSS statistical software (version 19.0).

## Results

The study sample consisted of 101 patients (69 men and 32 women) whose demographic and socioeconomic, baseline characteristics are presented in Table 1. More than half of the sample (64.3%) aged less than 35 years and only 25.3% had monthly income more than 500 euros. 61.4% of the sample characterized its economic status as bad or very bad, while 21.8% of the study participants were employed and 52.5% had a health insurance. With regards to physician visits 13.1% of the patients reported visiting their physician once a year, 4.0% once every six months, 7.1% once a month, and 1.0% more than once a month. The majority of the patients (74.7%) did not visit their practitioner at a regular basis but according to the practitioner’s instructions. Almost all patients (99.0%) visited physicians at public hospitals, while the remaining 1.0% visited practitioners at the health services affiliated to their social insurance fund.

**Table 1:** Sample demographic and socioeconomic characteristics

	N (%)
Sex	
Men	69 (68.3)
Women	32 (31.7)
Age (years)	
≤ 25	9 (8.9)
26–35	56 (55.4)
36–50	29 (28.7)
> 50	7 (6.9)
Place of residence	
Prefecture of Attica	101 (100.0)
Education	
Compulsory education	83 (83.8)
Secondary education	11 (11.1)
Higher education	3 (3.0)
Postgraduate education	2 (2.0)
Monthly income	
≤ 500	68 (74.7)
> 500	23 (25.3)
Self-assessment of economic status	
Good	1 (1.1)
Fair	33 (37.5)
Bad	17 (19.3)
Very bad	37 (42)
Employed	
Yes	22 (21.8)
No	79 (78.2)
Insurance	
Yes	53 (52.5)
No	48 (47.5)
Time from diagnosis (years), mean (SD)	5.8 (3.3)

Table 2 shows barriers to access treatment overall, and according to sex, monthly income, self-assessment of economic status and health insurance coverage. In total 86.9% of the participants reported experiencing difficulties in accessing their doctor during the past 12 months. The most common obstacles were “delay in making the appointment” (47.5%) and “difficulties in going to the doctor due to health condition or lack of means of transport” (46.5%). Barriers in accessing medication were reported by 84.8% of the subjects.

**Table 2:** Barriers to access treatment in total sample and according to demographic and socioeconomic determinants

	Total sample	Sex		Monthly income		Self-assessment of economic status		Health insurance	
		Males	Females	≤500	>500	Bad/Very bad	Good/Fair	No	Yes
	%	%	%	%	%	%	%	%	%
Barriers to physician access	86.9	83.6	93.8	82.4	95.7	81.1*	97.0	85.1	88.5
Delay in making appointment	47.5	50.7	40.6	39.7*	82.6	39.6	60.6	34.0*	59.6
Difficulties due to health reasons or lack of transportation means	46.5	41.8	56.3	50.0	26.1	50.9*	36.4	51.1	42.3
Language barriers	13.1	17.9	3.1	14.7	4.3	17.0	3.0	23.4*	3.8
Barriers to medication access	84.8	80.6	93.8	79.4	95.7	79.2*	97.0	80.9	88.5
Language barriers	14.1	16.4	9.4	14.7	4.3	17.0	3.0	23.4*	5.8

Note: Asterisks indicate significant difference in the proportions

Males and females had similar difficulties in accessing their physician and medication (*p* > 0.05). Patients with monthly income above 500 euro experienced barriers at a significantly greater percentage in making the appointment (*p* < 0.001). Participants with good/fair economic status had obstacles in accessing their doctor and medication at a significantly greater proportion compared to participants with bad/very bad economic status (*p* = 0.005 and *p* = 0.025 respectively). On the contrary, participants with good/fair economic status had difficulties in going to the doctor due to health reasons or lack of means of transport at a significantly lower percentage compared to participants with bad/very bad economic status (*p* = 0.046). Participants with health insurance coverage experienced long waiting lists at a significantly greater percentage compared to participants without health insurance (*p* = 0.011). On the other hand, participants without health insurance coverage faced language barriers in terms of accessing their doctor and medication at a significantly greater proportion compared to participants that had health insurance (*p* = 0.004 and *p* = 0.012 respectively).

**Fig. 1:**
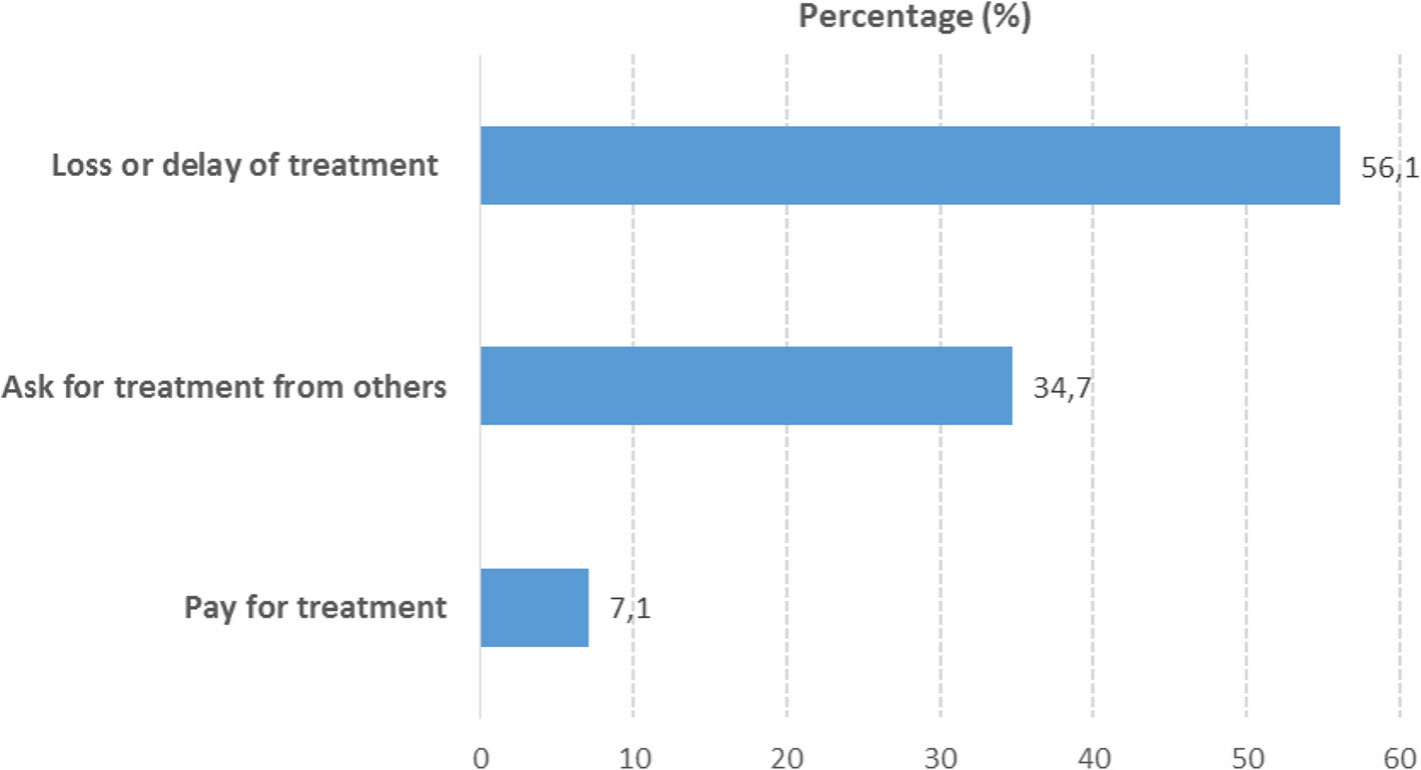
Consequences of barriers to access to HCV treatment

With regards to the consequences derived from barriers to access HCV treatment (Fig. 1) the most common was loss or treatment delay (56.1%), followed by request of treatment from other patients, NGOs etc. Also, 7.1% reported that they had to pay for their treatment.

**Fig. 2:**
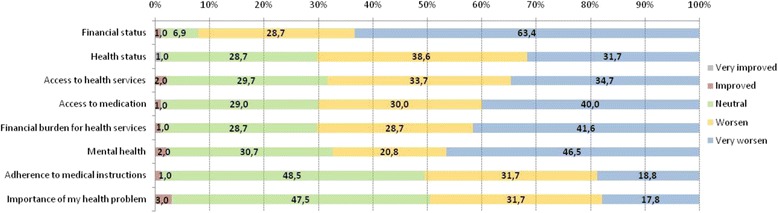
Assessment of current economical and health issues in comparison with 2009

Figure 2 presents the comparison of current economic and health status of the study participants with that of 2009. In more than half of the sample financial status, health status, access to health services, access to medication, financial burden for health services, mental health and adherence to medical instructions had worsened or very much worsened. Specifically, the financial status had worsened or had very much worsened in 92.1% of the cases, while the corresponding proportions for access to health services and access to medication were 68.4 and 70%, respectively.

Table 3 shows obstacles in accessing physician or medication in association with assessment of current economical and health issues, compared to 2009. Patients who had worse financial status compared to 2009 faced barriers in accessing physician and medication at a significantly greater percentage. Also, patients that reported worsened access to health services compared to 2009 experienced difficulties in accessing physician at a significantly greater proportion. Additionally, patients who reported increased financial burden for health services, lower adherence to medical instructions and worsened severity of their health problem in 2015 compared to 2009, also reported more obstacles in accessing physician and medication at a greater significance level.

**Table 3:** Barriers in treatment access and assessment of current economical and health issues compared to 2009

Compared to 2009 how would you describe your:	Obstacles in accessing their doctor		Obstacles in accessing their medication	
	N (%)	*P*	N (%)	*P*
Financial status				
Very improved to neutral	2 (25.0)	<0.001^a^	2 (25.0)	<0.001^a^
Worsen/Very worsen	84 (92.3)		82 (90.1)	
Health status				
Very improved to neutral	24 (82.8)	0.516^a^	23 (79.3)	0.326^a^
Worsen/Very worsen	62 (88.6)		61 (87.1)	
Access to health services				
Very improved to neutral	23 (74.2)	0.021^a^	23 (74.2)	0.068^a^
Worsen/Very worsen	63 (92.6)		61 (89.7)	
Access to medication				
Very improved to neutral	23 (79.3)	0.173^a^	23 (79.3)	0.342^a^
Worsen/Very worsen	63 (91.3)		61 (88.4)	
Financial burden for health services				
Very improved to neutral	18 (62.1)	<0.001^a^	18 (62.1)	<0.001^a^
Worsen/Very worsen	68 (97.1)		66 (94.3)	
Mental health				
Very improved to neutral	27 (84.4)	0.751^a^	26 (81.3)	0.553^a^
Worsen/Very worsen	59 (88.1)		58 (86.6)	
Adherence to medical instructions				
Very improved to neutral	36 (73.5)	<0.001^b^	35 (71.4)	<0.001^b^
Worsen/Very worsen	50 (100.0)		49 (98.0)	
Severity of my health problem				
Very improved to neutral	37 (74.0)	<0.001^b^	36 (72.0)	<0.001^b^
Worsen/Very worsen	49 (100.0)		48 (98.0)	

^a^Fisher’s exact test

^b^chi-square test

## Discussion

The present study aimed to investigate the barriers that injecting drug users are experiencing in accessing HCV treatment, as well as the possible associations between barriers to HCV treatment access and socioeconomic variables for the years 2009 and 2015. The study results indicated that the vast majority of IDUs experience significant difficulties in their effort to seek treatment for HCV. Specifically, IDUs of monthly income above 500 Euros, those who consider their socioeconomic status (SES) to be good/fair, as well as those with health insurance coverage reported barriers such as long waiting lists and problems in accessing medication at a higher significance level, compared to those with monthly income below 500 euros, those who reported bad/very bad SES, and those with no health insurance coverage. On the other hand, those of bad/very bad SES, seem to face more language barriers in accessing a physician and medication for HCV. These barriers were associated with the health status reported by IDUs in terms of treatment loss and/or delay and out-of-pocket cost for medication. It may seem contradictory that those of better SES experience more barriers in accessing physician and/or treatment compared with IDUs of low income and worse SES. In Greece, the health insurance coverage is linked with employment. Unemployed individuals do not have full medical care. At the same time, a health program covers people who leave in extreme poverty. As a result, insured persons who are employed follow the usual pathway within the healthcare system, while non-insured persons mainly seek help in non-governmental organizations and social and community services most of which are targeted to this population and thus have access in HCV test and treatment at no cost.

The vast majority of IDUs reported deteriorated financial status, health status, access to health services and access to medication, higher financial burden for health services, worse mental health and lower adherence to medical instructions in 2015 compared to 2009. Moreover, those of worse financial status as well as limited access to health services, increased financial burden for health services and lower adherence in 2015 compared to 2009, experienced more barriers in accessing physician and treatment.

Findings from the present study are in line with previous research efforts in the field. Non adherence, financial and social pressure, inadequate treatment availability and insufficient funding, have been highlighted as key barriers to HCV treatment for IDUs in many studies [3, 10, 33–38]. Depending on the legislation and the organization of the healthcare system in each country, the absence of health insurance coverage and the subsequent high out-of-pocket treatment cost is reported as a main inhibitor for HCV treatment by many researchers. It is noteworthy that, even though barriers exist also at patient level, 70–80% of IDUs assert that they are explicitly positive in receiving treatment for HCV [4].

The individuals who inject drugs and suffer HCV are lost during their journey in the healthcare system [10]. Apart from the barriers experienced by the general population, IDUs have to address additional difficulties in order to gain access to HCV treatment, such as the prerequisites for health insurance coverage, ensuring at least a basic income, avoid living as homeless and pursuit of a minimum of social support [39]. Apparently this process constitutes a difficult journey for IDUs. As a result, many IDUs who have been tested for HCV and are aware of the consequences of the disease, fail to follow a treatment plan effectively [37]. New treatments for HCV are highly effective, have less side-effects and do not require long time to show positive health outcomes [19]. On the other hand, many health systems and economies in European countries are in transition. New treatments for HCV do exist but they are not available to IDUs (regardless of whether an IDU is attending a rehabilitation program [3]. Research suggests that the key barriers to HCV treatment for IDUs are related to the healthcare system and the social norms imply that IDUs are responsible for the damage caused to their health [25]. Criticizing a person for injecting drugs or having a co-occurring psychiatric condition is known in health promotion as “blame the victim” [40]. Misconceptions and stigmatization for IDUs result in an unfavorable situation for their health and quality of life. It is estimated that approximately 72% of IDUs in Europe do not receive treatment, even though they have been tested for HCV, due to the abovementioned barriers [41]. It should be noted that heroin use does not affect patient response and adherence to HCV treatment in contrast with alcohol abuse which may aggravate the effectiveness of HCV medication [19, 28].

The outbreak of the recession in 2008 had a significant effect not only on the economy but also on the health of the population in many European countries. In Spain, increased drug and injection drug use in long-term unemployed and people living below the poverty line is highlighted and associated with increased inequalities [41]. Some countries perform better than others. In Portugal and United Kingdom for example inequalities were decreased while in others such as Spain and Greece were increased. Inequalities affect disproportionally the health of vulnerable groups which in turn are at increased risk for drug use and abuse, communicable and non-communicable diseases, as well as limited access to the healthcare system [14].

Previous studies highlighted that the health inequalities to IDUs are mainly attributed to key-factors such as homelessness, socioeconomic status and incarceration which also considered as social determinants of health [42]. The researchers emphasize that the risk behaviour itself is not responsible for the unequal morbidity and mortality in this particular group, but this unfavorable situation is better interpreted by the impact of the social determinants of health [43]. The social environment of IDUs is abounded of deprivations that shape their overburdened health future. For instance, the association of SES with health status is well established. Individuals of low SES are at significantly increased risk to suffer from non communicable diseases such as heart disease [44], have worst quality of life [45] and die at a younger age [46]. The social gradient is strongly linked to the morbidity and mortality rates not only in IDUs but also for the general population [47, 48]. Evidence suggests that as the SES is increased the health risk is decreased and vice versa [48]. If we want to gain a deeper understanding about the impact of social determinants of health on IDUs who suffer from HCV we have to consider every level of the societal influence on their health. IDUs have more possibilities to grow-up and continue to live in poverty and of high crime rate neighborhoods, with fewer opportunities to have a job and health insurance coverage, with limited or absent social support [43]. As a result they live marginalized in the boundaries of their community and have little chance of access to HCV treatment, to drug-addiction services and finally little prospect to live a healthy and prosperous life [49]. Of course in the context of socio-ecologic model, broader contexts such as the effectiveness of the health system and the social policies implemented are also affecting this group [50]. Thus, we should consider these effects as direct and indirect on IDUs health. For example, the horizontal budget cuts influencing indirectly IDUs health (due to the deficiencies in the health system) while extreme poverty has a direct impact [43, 51].

Inequalities do exist within and between countries [50]. In Greece there is an ongoing economic crisis since 2009, and the GDP has been reduced substantially and significantly more compared to other European countries. Evidence shows that GDP reduction between 2008 and 2012 was negatively associated with the reporting rate of IDU, and HCV and HVI co-infection, as well as with the unemployment rate and homelessness [26]. Congruent with these, findings from the present study indicate that in 2015, IDUs reported worse financial and health status, decreased access to health services and medication, higher financial burden for health services, and lower adherence to medical instructions compared to 2009. In other words, instead of strengthening the protective factors of this vulnerable group, the risk factors were enhanced and the possibilities of premature mortality were increased [26].

## Study limitations

Results in the present study should be considered in the light of the following limitations. The assessment of the IDUs’ financial status was based on self-reported measurements. This implies that an over or under-estimation of the socioeconomic level is possible. This is a common limitation in most studies on this population. Most researchers fail to assess the SES of IDUs and they inadequately evaluate their social standing. An additional limitation is the weakness of the cross-sectional studies to ascertain causal relationships. Consistent with this, we cannot claim that the consequences of the financial crisis in Greece are responsible for the limited access of IDUs to HCV treatment and vice versa. Moreover, we cannot assume that IDUs were at increased risk for limited access before the financial crisis and the ongoing recession acted as a trigger to worsened health status and job loss (reverse causation). Limitations also apply due to the use of convenient sampling.

## Conclusion and policy implications

The present study showed that the vast majority of IDUs experience significant barriers in seeking HCV care, thus highlighting the need for action in this particular area.

In the current financial circumstances in Greece and other European countries, it is expected that reforms should be implemented and health services need to be re-oriented in order to meet the health needs of the vulnerable groups and not just implement policies such as reduced public spending on health and healthcare, as a response to the recession. Policy makers often employ budget cuts as an instant and reliable measure in financial terms in order to contain costs, but this does not constitute a sustainable policy in cost-effectiveness, health promotion and well- being terms. The research and health community should advocate for vulnerable groups such as IDUs suffering from HCV in order to increase awareness about inequalities in health and accelerate actions on specific areas. It is crucial to call for accountability and support action in the following priority areas in order to improve the access to HCV treatment for IDUs:

- ▪ Raise awareness about stigmatization of IDUs among health professionals
- ▪ Develop, implement and evaluate multi-faceted programs (e.g. rapid screening for HCV, provision of opioid substitution therapy, increased access to HCV treatment).
- ▪ Enhance expertise and facilitate the exchange of best practices among European countries.

## Abbreviations

EU: European Union
HCV: Hepatitis C virus
Hep-B: Hepatitis B
HIV: Human Immunodeficiency Virus
HOPE: Health Outcomes of Patient Environment
IDU: Injection drug use
IDUs: Injecting drug users
NGO: Non-Governmental Organization
SES: Socio- Economic Status
